# Heat Transfer and Temperature Characteristics of a Working Digital Camera

**DOI:** 10.3390/s20092561

**Published:** 2020-04-30

**Authors:** Shichao Zhou, Haibin Zhu, Qinwei Ma, Shaopeng Ma

**Affiliations:** 1 School of Aerospace Engineering, Beijing Institute of Technology, Beijing 100081, China; zhoushichao1234@bit.edu.cn; 2 Flexible Optical Measurement Technology Center, Institute of Flexible Electronic Technology of THU, Jiaxing 314006, China; zhuhaibin@ifet-tsinghua.org; 3 School of Naval Architecture, Ocean & Civil Engineering, Shanghai Jiao Tong University, Shanghai 200240, China; mashaopeng@sjtu.edu.cn

**Keywords:** digital camera, heat transfer, temperature characteristics

## Abstract

Digital cameras represented by industrial cameras are widely used as image acquisition sensors in the field of image-based mechanics measurement, and their thermal effect inevitably induces thermal-induced errors of the mechanics measurement. To deeply understand the errors, the research for digital camera’s thermal effect is necessary. This study systematically investigated the heat transfer processes and temperature characteristics of a working digital camera. Concretely, based on the temperature distribution of a typical working digital camera, the heat transfer of the working digital camera was investigated, and a model describing the temperature variation and distribution was presented and verified experimentally. With this model, the thermal equilibrium time and thermal equilibrium temperature of the camera system were calculated. Then, the influences of thermal parameters of digital camera and environmental temperature on the temperature characteristics of working digital camera were simulated and experimentally investigated. The theory analysis and experimental results demonstrate that the presented model can accurately describe the temperature characteristics and further calculate the thermal equilibrium state of working digital camera, all of which contribute to guiding mechanics measurement and thermal design based on such camera sensors.

## 1. Introduction

For common sensor systems, thermal effect, in general, occurs in many different practical applications and in different forms, due to their self-heating and temperature variation of surroundings. Considering the performance of such sensors, especially high-precision sensor systems, the thermal effect plays a very important role [1,2,3,4,5]. On the one hand, thermal effect may cause adverse impact on the reliability of sensors and even result in their function failure [1,2]; on the other hand, for common temperature-sensitive sensors, thermal effect can reduce the accuracy of their measurement results [3,4,5]. Mainly driven by complexity of application environment, reliability enhancement and implementation of high-accuracy measurement, the investigations of thermal properties of materials [6,7,8,9,10,11,12], thermal design, and control of sensor systems [13,14,15,16], as well as thermal compensation techniques [17,18], have been extensively carried out. It is worth noting that whether the evaluation of the adverse impacts on sensor systems and its measurement accuracy induced by thermal effect or developments of the methods and techniques to eliminate such adverse impacts, it is critical to understand the heat transfer and temperature characteristics of these sensors [11,12,14,19,20,21].

Digital camera is a kind of important photoelectric sensor for image acquisition, such as widely used industrial camera in image-based mechanics measurements [22] with internal heating source and multiple components, which include camera case, mount for mechanically connecting lens, and optical lens. As a precision and temperature-sensitive sensor, the investigation of these digital camera’s thermal effect is vital and imperative. In general, when the digital camera works, the interaction of the heat generated by the internal heating source and environmental temperature causes the temperature variation of the whole camera system [23,24,25], and further thermal deformations of the multiple components [23,25,26,27]. Even though such thermal deformations are very slight, there are significant adverse impacts on imaging optical path, i.e., imaging parameters, and further measurement errors in mechanics [23,24,25,26,27,28,29,30,31]. Based on this matter, some researchers carried out investigations on the compensation of thermal-induced errors in mechanics measurement [23,24,25,32,33,34]. These investigations, however, focus on the relationship between imaging parameters variation and the measurement errors [32,33] or the relationship between thermal deformation of camera components and the measurement errors [23], but are shallow for the investigation of digital camera’s temperature variation as the fundamental reason of the measurement errors. In fact, the temperature variation characteristics of digital camera play an important role in the investigation of the thermal-induced errors in image-based mechanics measurement. Yu et al. [33] pointed out that the variations in the camera imaging parameters depend on the variations in camera temperature, and the main influencing factors include the camera’s absolute temperature, the value of temperature variation and the velocity of the temperature variation. In this regard, the temperature compensation method [23], which uses the camera case’s temperature to characterize the temperature of the whole camera system, can only be applied to the compensation of the thermal-induced error caused by camera self-heating, that is to say, it is not applicable to the error correction in the case of complex temperature variations. Therefore, to develop temperature compensation methods suitable for complex temperature changes, it is necessary to study the temperature characteristics of a working digital camera system. In addition, the references [23,24,28,31] shown that when the image-based mechanics measurement is performed with a working digital camera in thermal equilibrium, there will be no thermal error. Hence, via studying the camera’s temperature variation characteristics to determine the thermal equilibrium state of the camera, i.e., thermal equilibrium time and thermal equilibrium temperature, the time period of performing the mechanics measurement without thermal-induced error, named ‘measurement time window’, can be effectively determined.

So far, these existing methods for obtaining temperature data of digital camera are usually based on the measurement of temperature variation at one [26,28,31] or multiple locations [27,30,32,33] of the camera system, and the camera system’s temperature filed detection using thermal infrared camera [23,24], all of which cannot reveal the mechanism of temperature characteristics of the whole camera system over time, which limits the applicability of the existing compensation methods in complex thermal environments. In fact, considering the coaction of time-varying environmental temperature and camera self-heating, the temperature characteristics of a working camera system present complexity. Firstly, the temperature variation of camera system is complex [25], and the relationships between camera components temperature are no longer simply linear function just considering camera self-heating [23]. Secondly, the temperature distribution of camera components exhibits heterogeneity, which depends on the structure, material and size of those components. To date, there is no research reports to describe the complexity of temperature variation and the heterogeneity of temperature distribution in digital camera system with the analysis of heat transfer under the coaction of time-varying environmental temperature and camera self-heating. Note that for both the development of temperature compensation methods in image-based mechanics measurement and the implementation of digital camera’s thermal design, it is necessary to investigate heat transfer of the whole camera system, and further to establish model describing the temperature characteristics of such system.

In this study, therefore, the heat transfer of a working digital camera system was investigated under the coupling effect between camera self-heating and environmental temperature, and a physical model was presented to describe the temperature variation and distribution of the digital camera system. With the model, the temperature of a digital camera system was calculated via environmental temperature. Then, the temperature characteristics of digital camera with different thermal parameters and different environmental temperatures were investigated by simulation and experiment.

## 2. Model for Heat Transfer Process of Working Digital Camera Systems

### 2.1. Temperature Distribution of Working Typical Camera Systems

**Figure 1:**
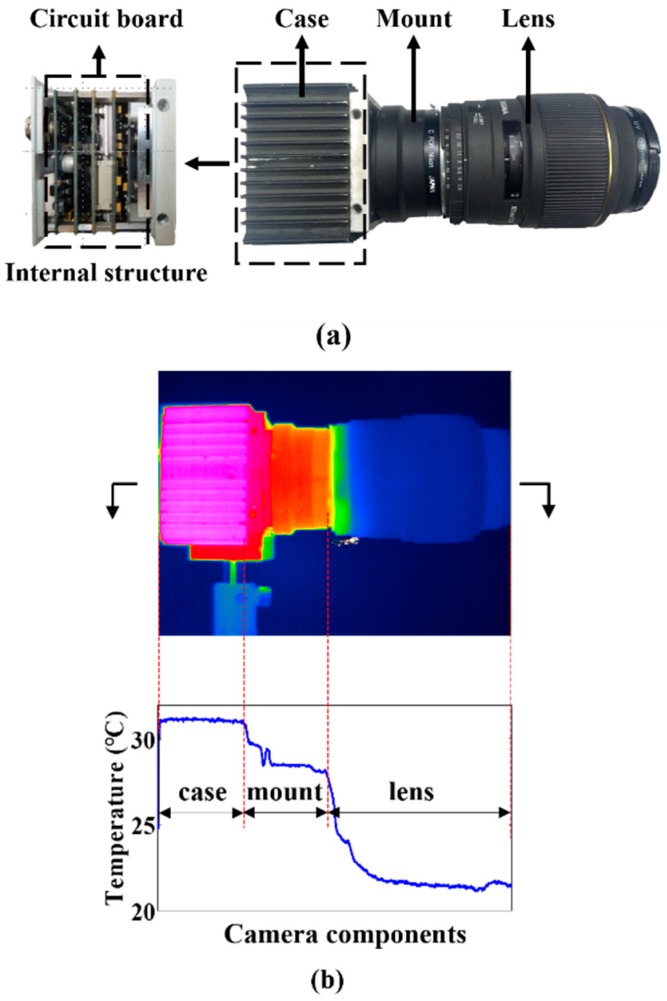
(**a**) Structural composition and (**b**) temperature distribution of typical digital camera system monitored by infrared camera.

As illustrated in Figure 1a, a typical digital camera system is mainly composed of the camera case, integrated circuit board (the heating source during camera operation), the mount, and the lens. When the camera is operating, heat generated by the circuit board will be transferred between the camera’s mechanical components and the environment, all of which leads to the temperature variation of the components. The integrated circuit boards are approximately uniformly distributed in the camera case, which will result in uniform temperature distribution of camera case. In addition, since the mount and lens can be considered as axisymmetric cylinders in terms of structure and material composition, the temperature distribution of the mount and lens should also show axisymmetric characteristics. Figure 1b shows the temperature distribution of the components after the camera system reaches thermal equilibrium. The temperature distributions of the camera components indicate that the temperature distribution of the case is uniform; the temperature distributions of the mount and lens are non-uniform. The closer to the heat source, the higher the temperature. All of which match the above analysis of the camera-components temperature characteristics. Therefore, this study assumes that the heat transfer of the camera is one-dimensional.

### 2.2. Temperature Model of Working Digital Camera System

**Figure 2:**
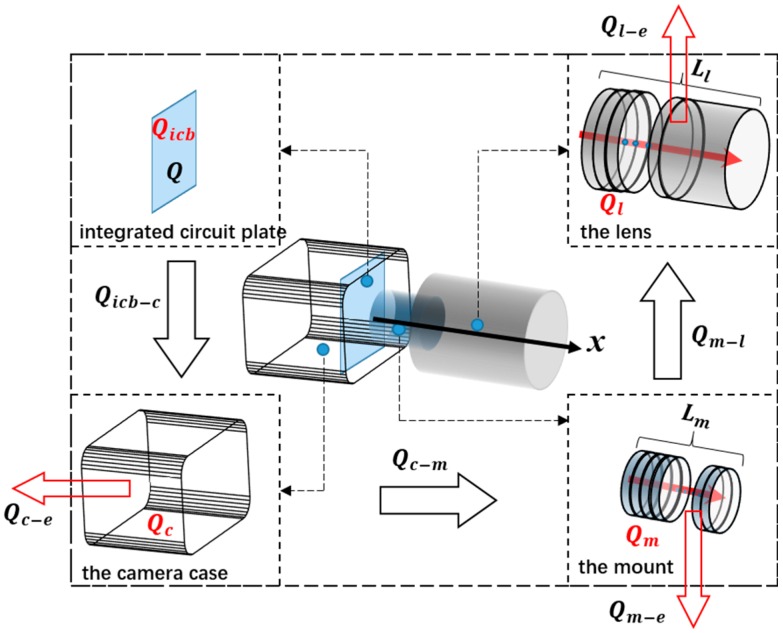
Heat transfer path of a working digital camera system.

According to the composition of the digital camera system, the entire heat transfer process can be classified into three components: heat conduction between the integrated circuit board, case, mount, and lens; heat convection between the camera’s mechanical components and the environment; heat absorption of the camera components. The heat transfer path of the digital camera system under the coupling effect of self-heating and environmental temperature is shown in Figure 2.

The heat generated by the integrated circuit board is assumed to be *Q*, a part of which (*Q_icb_*) changes the temperature of the integrated circuit board, and the rest (*Q_icb−c_*) transfers to the camera case. *Q_icb−c_* can be further divided into three components: *Q_c_* changes the temperature of the camera case, and *Q_c−e_* is exchanged between the case and the environment, and *Q_c−m_* that transfers to the mount. *Q_c−m_* can also be divided into three components: *Q_m_* that changes the temperature of the mount, and *Q_m−e_* is exchanged between the mount and the environment, and *Q_m−l_* that transfers to the lens. Finally, *Q_m__−l_* can be divided into two components: *Q_l_* that changes the temperature of the lens, and *Q_l__−e_* is exchanged between the lens and the environment. Thus, the heat transfer process can be described by the expressions:

(1) $$\left\{ \begin{array}{l} Q=Q_{icb}+Q_{icb-c}\\ Q_{icb-c}=Q_{c}+Q_{c-e}+Q_{c-m}\\ Q_{c-m}=Q_{m}+Q_{m-e}+Q_{m-l}\\ Q_{m-l}=Q_{l}+Q_{l-e} \end{array}\right.$$

Combined with the fundamental formulae of thermology, including Fourier’s law of heat conduction, Newton’s cooling formula, and the specific heat capacity formula [19], Equation (1) can be rewritten as:

(2) $$\left\{ \begin{array}{l} P-R_{1}(T_{icb}-T_{c})=K_{1}\frac{dT_{icb}}{dt}\\ R_{1}(T_{icb}-T_{c})-R_{2}(T_{c}-T_{e})-R_{3}(T_{c}-T_{m}|{}_{L_{m}})=K_{2}\frac{dT_{c}}{dt}\\ R_{3}(T_{c}-T_{m}|{}_{L_{m}})-\int_{0}^{L_{m}}R_{4}(T_{m}-T_{e})dx-R_{5}(T_{m}|{}_{L_{m}}-T_{l}|{}_{L_{l}})=\int_{0}^{L_{m}}K_{3}\frac{\partial T_{m}}{\partial t}dx\\ R_{5}(T_{m}|{}_{L_{m}}-T_{l}|{}_{L_{l}})-\int_{L_{m}}^{L_{m}+L_{l}}R_{6}(T_{l}-T_{e})dx=\int_{L_{m}}^{L_{m}+L_{l}}K_{4}\frac{\partial T_{l}}{\partial t}dx \end{array}\right.$$

where *P* denotes the thermal power (units: W) of the integrated circuit board. *R*_1_, *R*_3_, and *R*_5_ represent the heat conduction parameters (units: W/°C) of the case, mount, and lens, respectively, and are directly proportional to the material’s thermal conductivity, cross-sectional area, and inversely proportional to the length of the heat conduction direction of the corresponding components. *R*_2_, *R*_4_, and *R*_6_ represent the heat convection parameters (units: W/°C) of the case, mount, and lens, respectively, and are directly proportional to the material’s surface heat transfer coefficient and superficial area of the corresponding components. *K*_1_–*K*_4_ denote specific heat parameters (units: J/°C) of the integrated circuit board, case, mount, and lens, respectively, and are directly proportional to the material’s specific heat capacity and quality of the corresponding components. *T_icb_*, *T_c_*, and *T_e_* are the temperature of the integrated circuit board, case, and environment, respectively, all of which are the function of time *t*. *T_m_* and *T_l_* are the temperature of the mount and lens, respectively, all of which are the function of time *t* as well the position coordinate *x* whose direction along the optical axis and origin is located on the contact surface between the mount and the case. *L_m_* and *L_l_* represent the length of the mount and lens along the optical axis, respectively.

Equation (2) expresses the relationship between the camera component temperatures (*T_c_*, *T_m_*, and *T_l_*), the environmental temperature (*T_e_*), and the thermal power (*P*) of the working digital camera. The temperature of the camera components cannot be solved first-hand according to Equation (2); to calculate the camera components temperatures, Equation (2) is firstly simplified to obtain the temperature of the case (*T_c_*), the boundary temperature of the mount (${T_{m}|}_{L_{m}}$), and the boundary temperature of the lens (${T_{l}|}_{L_{l}}$). Then, based on analysis of the internal heat transfer of the mount (lens), the temperature expression of the mount (lens) is established. The simplification of Equation (2) is as follows:

(3) $$\left\{ \begin{array}{l} 1-r_{1}(T_{icb}-T_{c})=k_{1}\frac{dT_{icb}}{dt}\\ r_{1}(T_{icb}-T_{c})-r_{2}(T_{c}-T_{e})-r_{3}(T_{c}-T_{m}|{}_{L_{m}})=k_{2}\frac{dT_{c}}{dt}\\ r_{3}(T_{c}-T_{m}|{}_{L_{m}})-r_{4}(T_{m}|{}_{L_{m}}-T_{e})-r_{5}(T_{m}|{}_{L_{m}}-T_{l}|{}_{L_{l}})=k_{3}\frac{dT_{m}|{}_{L_{m}}}{dt}\\ r_{5}(T_{m}|{}_{L_{m}}-T_{l}|{}_{L_{l}})-r_{6}(T_{l}|{}_{L_{l}}-T_{e})=k_{4}\frac{dT_{l}|{}_{L_{l}}}{dt} \end{array}\right.$$

where *r*_1_, *r*_3_, and *r*_5_ are the characteristics heat conduction parameters of the case, mount, and lens, respectively; *r*_2_, *r*_4_, and *r*_6_ are the characteristics heat convection parameters of the case, mount, and lens, respectively; and *k*_1_–*k*_4_ are the characteristics specific heat parameters of the integrated circuit board, case, mount, and lens, respectively. The above-defined parameters are related to the thermal power of working digital camera. If the abovementioned parameters are obtained, the temperature of the case (*T_c_*), the boundary temperature of the mount (${T_{m}|}_{L_{m}}$), and the boundary temperature of the lens (${T_{l}|}_{L_{l}}$) can be calculated via the environmental temperature.

**Figure 3:**
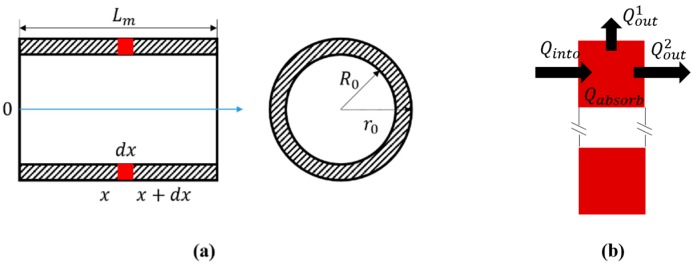
Analysis of heat transfer for the mount: (**a**) cross section of mount, (**b**) the heat transfer path of the *dx*.

Next, the temperature expressions of the mount and lens are established. The shape of the mount can be simplified to a cylinder with inner diameter *R*_0_, outer diameter *r*_0_, and length *L*_m_. Figure 3a shows cross sections of the simplified mount along the optical axis and perpendicular to the optical axis. The heat transfer path of object *dx* is shown in Figure 3b; according to the conservation of energy, the heat transfer can be expressed as:

(4) $$Q_{\mathrm{int}o}=Q_{out}^{1}+Q_{out}^{2}+Q_{absorb}$$

where *Q_into_* is the heat flowing into *dx* from the last infinitesimal; $Q_{out}^{1}$ is the heat flowing into the environment from *dx*; $Q_{out}^{2}$ is the heat flowing into the next infinitesimal from *dx*; and *Q_absorb_* is the heat increment of *dx*.

Combined with the fundamental formulae of thermology, including Fourier’s law of heat conduction, Newton’s cooling formula, and the specific heat capacity formula [19], Equation (4) can be rewritten as:

(5) $$k_{m}\frac{\partial^{2}T_{m}}{\partial x^{2}}=m_{m}(T_{m}-T_{e})+n_{m}\frac{\partial T_{m}}{\partial t}$$

where *k_m_*, *m_m_*, and *n_m_* represent the heat conduction parameter, the heat convection parameter, and the specific heat parameter of the mount, respectively. If those parameters are obtained, the temperature of the mount (*T_m_*) can be calculated via the environmental temperature, the initial temperature, and the boundary temperature of the mount.

Similarly, the temperature expression of the lens can be expressed as:

(6) $$k_{l}\frac{\partial^{2}T_{l}}{\partial x^{2}}=m_{l}(T_{l}-T_{e})+n_{l}\frac{\partial T_{l}}{\partial t}$$

In summary, Equation (2) describes the temperature variation and distribution of a working digital camera system, which can be calculated by Equations (3), (5), and (6). Thus, the temperature model is established.

The thermal equilibrium time and thermal equilibrium temperature during the working process of a camera system are important values in mechanics measurement. In this paper, the thermal equilibrium time refers to the time period during which the temperature of working camera remains constant and the thermal equilibrium temperature refers to the camera’s temperature during the thermal equilibrium time. The precise value of the thermal equilibrium time and temperature can effectively guide the selection of the ‘measurement time window’ so as to eliminate thermal-induced errors of mechanics measurement. After the working camera system reaches thermal equilibrium, the temperature variation of any point (*i*) of the camera satisfies:

(7) $$\left\{ \begin{array}{l} \frac{dT_{i}}{dt}=0\\ \frac{\left|\frac{d^{2}T_{i}}{dt^{2}}\right|}{{\left[1+{\left(\frac{dT_{i}}{dt}\right)}^{2}\right]}^{3/2}}=0 \end{array}\right.$$

In other words, the slope and curvature of the temperature curve over time are all zero for any measurement point on the camera system, all of which can be calculated by differential algorithms.

### 2.3. Experimental Verification

In this subsection, taking the digital camera system consisting of an IPX-16M3-L CCD camera and Sigma DG 28-300 mm lens, the accuracy of the camera component’s temperature variation and distribution calculated using the temperature model was verified experimentally. Firstly, in order to obtain the specific temperature model, the thermal parameters in Equations (3), (5), and (6) were calibrated by the optimization algorithm of nonlinear least square (the MATLAB’s built-in nonlinear least square function, i.e., lsqnonlin). Then, under the coaction of camera self-heating and time-varying environmental temperature, the temperatures of the camera components were calculated via the above obtained temperature model, and compared to the measured camera component temperatures measured using thermal sensors (i.e., the reference values for experimental verification). Finally, based on the verified temperature model, the thermal equilibrium time and thermal equilibrium temperature of the working digital camera were calculated.

**Figure 4:**
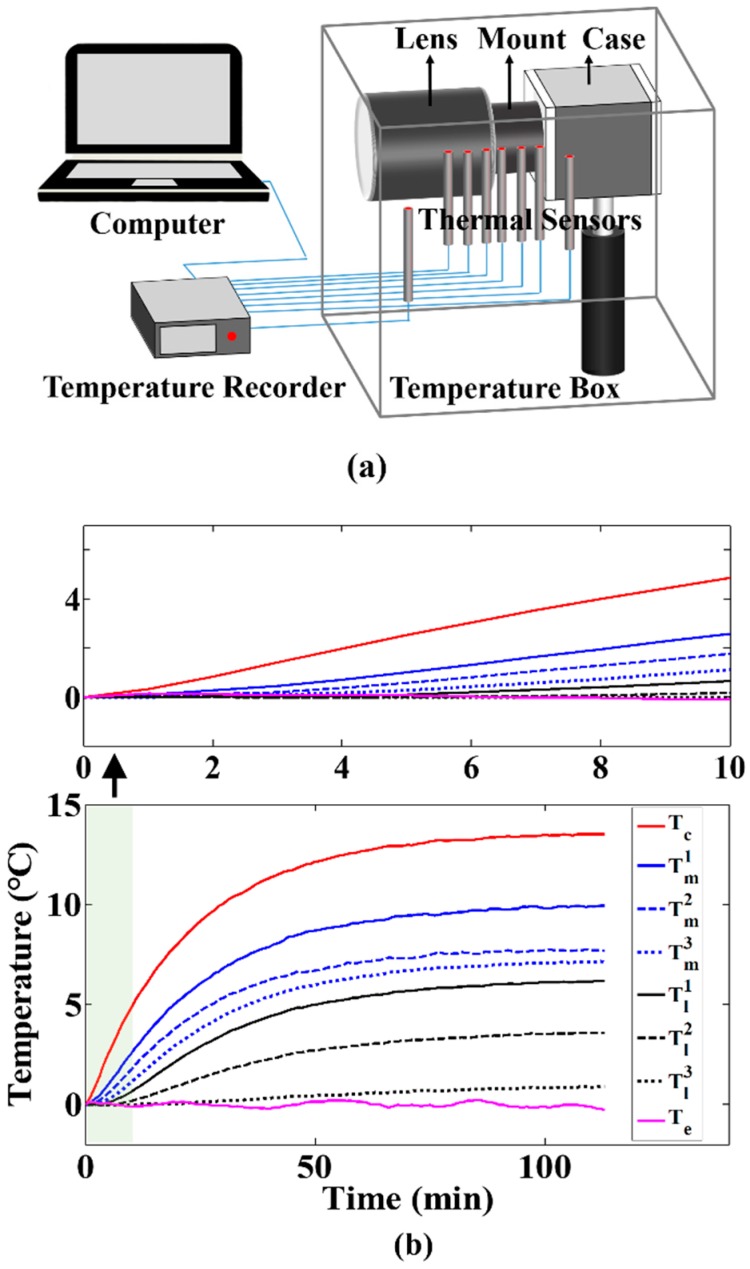
Parameter calibration of the temperature model: (**a**) schematic of the experimental setup, (**b**) temperature variation of camera components.

First, the obtaining of specific temperature model is introduced in detail. The thermal parameters of the camera system were calibrated using the camera component temperature variation value induced by camera self-heating. A schematic and layout of the calibration-experiment setup are shown in Figure 4a. The camera system was placed in a temperature box (an electric heating equipment named CINITE MAC3) that achieved the constant environmental temperature. The temperature data of the camera case, the different measurement points of the mount, the different measurement points of the lens and environment were measured using thermal sensors (K-type thermocouple, precision of 0.01 °C) during the process of camera self-heating. The experimental results are shown in Figure 4b. In the whole process, the average value and variance of environmental temperature are −0.01 and 0.01 °C, respectively. Therefore, the influence of environmental temperature in those thermal parameters’ calibration was ignored.

According to the experimental temperature data of the case (*T_c_*), the boundary of the mount ($T_{m}^{3}$), and the boundary of the lens ($T_{l}^{3}$), the thermal parameters of Equation (3) were obtained via the optimization algorithm; the results are given in Table 1. According to the measured temperature data of the mount ($T_{m}^{1}$, $T_{m}^{2}$, and $T_{m}^{3}$) and assuming that *n_m_* = 1.00, the thermal parameters (*k_m_*, *m_m_*) of Equation (5) were obtained via the optimization algorithm; the results are given in Table 2. According to the measured temperature data of the lens ($T_{l}^{1}$, $T_{l}^{2}$, and $T_{l}^{3}$) and assuming that *n_l_* = 1.00, the thermal parameters (*k_l_*, *m_l_*) of Equation (6) were obtained via the optimization algorithm; the results are given in Table 3. The specific relationship between the environmental temperature and the camera components temperature was determined by substituting the calibration results into Equations (3), (5), and (6).

**Figure 5:**
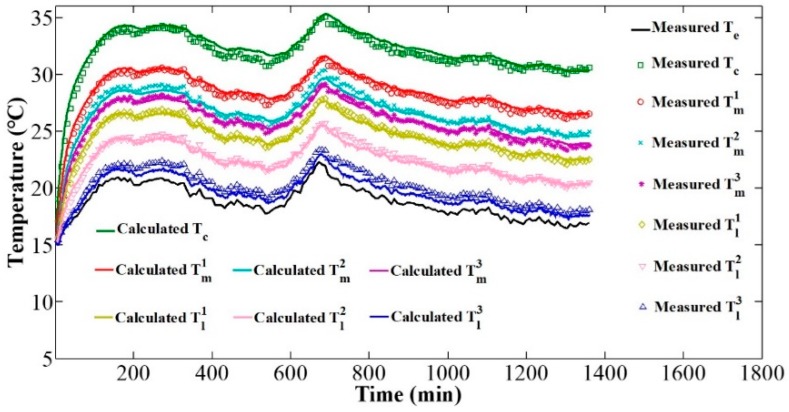
Temperature variation of the measured and calculated results over time.

**Figure 6:**
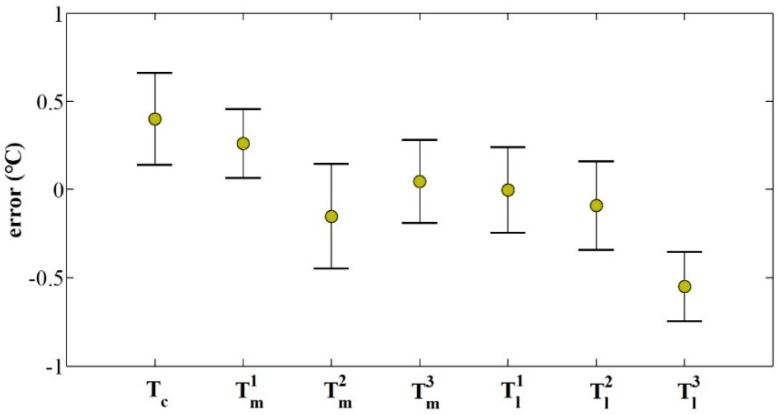
Errors between measured and calculated temperatures: the marker represents the average error, which ranges from −0.6 to 0.5 °C; the error bar represents the variance of the error, which ranges from 0.2 to 0.3 °C.

Next, the validity of the temperature model was verified experimentally with the coaction of camera self-heating and time-varying environmental temperature. The layout of the verification experiment is the same as shown in Figure 4. During the process of camera self-heating, the environmental temperature controlled by the temperature box changed over time. The environmental temperature and the camera component temperatures were measured by thermal sensors. According to the measured environmental temperature and the obtained temperature model, the theoretical camera component temperatures were calculated, and compared to the measured temperature of the corresponding components. Figure 5 shows the variation of the measured camera component temperatures and the calculated temperatures over the whole verification experiment. Figure 6 shows the errors between the calculated temperatures and the measured temperatures over the whole experimental process. The average errors range from just −0.6 to 0.5 °C and the variances range from 0.2 to 0.3 °C, confirming the accuracy of the proposed temperature model to describe camera component temperature variations and distribution.

**Figure 7:**
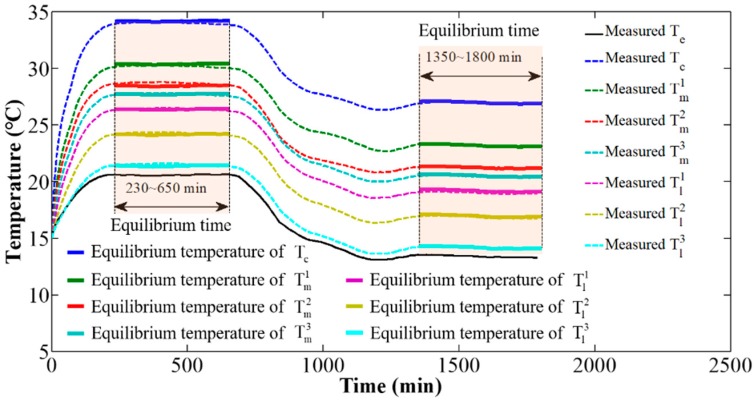
Calculated thermal equilibrium time and equilibrium temperature: in the first equilibrium time (230 to 650 min), the equilibrium temperatures of the target points on the camera are 34.2, 30.4, 28.4, 27.7, 26.4, 24.2, 21.4 °C, respectively; in the second equilibrium time (1350 to 1800 min), the equilibrium temperatures are 27.0, 23.2, 21.3, 20.5, 19.2, 17.0, 14.2 °C, respectively.

Finally, the thermal equilibrium time and thermal equilibrium temperature were investigated via the temperature model. By controlling the temperature box, the environmental temperature firstly rose to nearly 20 °C, then maintained for a period of time, and finally gradually decreased to a stable state. During the experiment, the camera component temperatures and environmental temperature were measured by means of thermal sensors. Through the measured environmental temperature, the temperature model, Equation (7), in which the threshold of slope and curvature were set to 0.001, the thermal equilibrium time, and the equilibrium temperature were calculated, as shown in Figure 7. The experimental results confirm that our temperature model can accurately calculate the thermal equilibrium time and equilibrium temperature. Once the camera’s thermal equilibrium state is obtained, the time period of performing the mechanics measurement without thermal-induced error can be effectively determined.

## 3. Temperature Variation and Distribution

In order to fully understand the temperature characteristics of digital camera under different thermal parameters and different environmental temperature, simulation and experimental investigations are necessary with the abovementioned presented temperature model. Concretely, under a constant environmental temperature, the influences of heat conduction, heat convection, and specific heat on the camera component temperature variations and distributions were studied. Then, under constant thermal parameters, the influence of environmental temperature on the temperature variations and distributions was investigated and verified experimentally.

### 3.1. Influence of Thermal Parameters

The thermal parameters in Equations (3), (5), and (6) can be divided into three categories: heat conduction parameters, including *r*_1_, *r*_3_, *r*_5_, *k_m_*, and *k_l_* are used to describe the performance of internal heat transfer in camera components; heat convection parameters, including *r*_2_, *r*_4_, *r*_6_, *m_m_*, and *m_l_* are used to describe the performance of heat transfer between camera components and the environment; specific heat parameters, including *k*_1_, *k*_2_, *k*_3_, *k*_4_, *n_m_*, and *n_l_* are used to describe the ability to temperature variation of those camera components. When the influence of one type of parameter on the temperature variation and distribution was studied, the other two parameters types were set to the values given in Table 1, Table 2 and Table 3.

**Figure 8:**
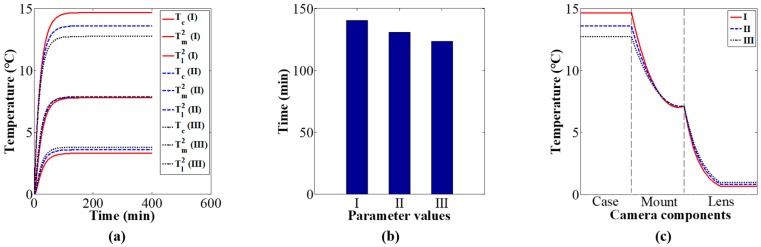
Effect of heat conduction parameters on camera component temperature variation and distribution: (**a**) components temperature variation, (**b**) thermal equilibrium time, and (**c**) distribution of equilibrium temperature.

Using the values of the heat conduction parameters in Table 4, the influence of heat conduction on the camera component temperature variations and distributions was studied via simulation. Figure 8a shows camera component temperature variation curves over time under different values of the heat conduction parameters. The simulation results indicate that the thermal equilibrium time of camera components decrease with the increase in the heat conduction parameters, as shown in Figure 8b; the distribution of the thermal equilibrium temperature become more uniform with the increase in the heat conduction parameters, as shown in Figure 8c.

**Figure 9:**
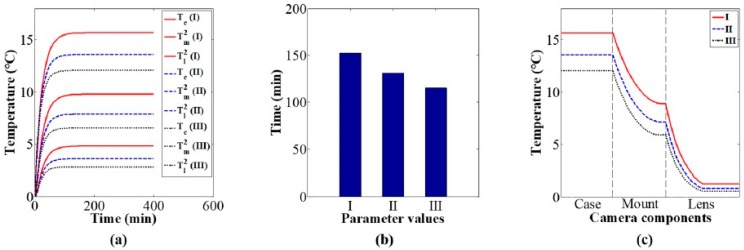
Effect of heat convection parameters on camera component temperature variation and distribution: (**a**) components temperature variation, (**b**) thermal equilibrium time, and (**c**) distribution of equilibrium temperature.

Based on the values of the heat convection parameters in Table 5, the influence of heat convection on the camera component temperature variations and distributions was studied via simulation. Figure 9a shows camera component temperature variation curves over time under different values of the heat convection parameters. The simulation results indicate that the thermal equilibrium time of the camera components decrease with the increase in the heat convection parameters, as shown in Figure 9b; the thermal equilibrium temperature decrease with the increase in the heat convection parameters, as shown in Figure 9c.

**Figure 10:**
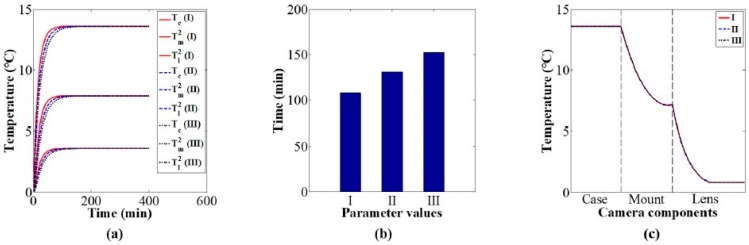
Effect of specific heat parameters on camera component temperature variation and distribution: (**a**) components temperature variation, (**b**) thermal equilibrium time, and (**c**) distribution of equilibrium temperature.

Based on the values of the specific heat parameters in Table 6, the influence of specific heat on the camera component temperature variations and distributions was studied via simulation. Figure 10a shows camera component temperature variation curves over time under different values of the specific heat parameters. The simulation results indicate that the thermal equilibrium time of camera components increase with the increase in the specific heat parameters, as shown in Figure 10b; the thermal equilibrium temperature is almost constant for different values of the specific heat parameters, as shown in Figure 10c.

The above investigations indicate that under a constant environmental temperature, thermal equilibrium time is negatively correlated with both heat conduction parameters and heat convection parameters, and positively correlated with specific heat parameters. The distribution of thermal equilibrium temperatures is affected by heat conduction parameters and convection parameters, and it is independent of specific heat parameters.

### 3.2. Influence of Environmental Temperature

**Figure 11:**
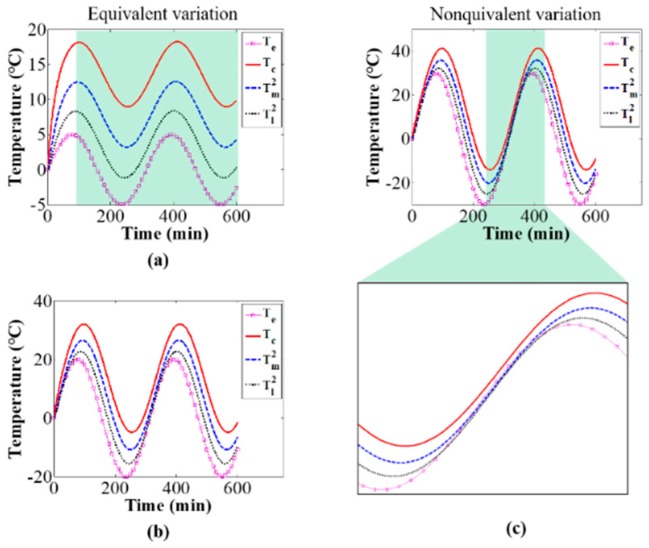
Effect of environmental temperature variation rate on camera components temperature: (**a**) $T_{e}=5\sin(t/50)$, (**b**) $T_{e}=20\sin(t/50)$, and (**c**) $T_{e}=30\sin(t/50)$.

**Figure 12:**
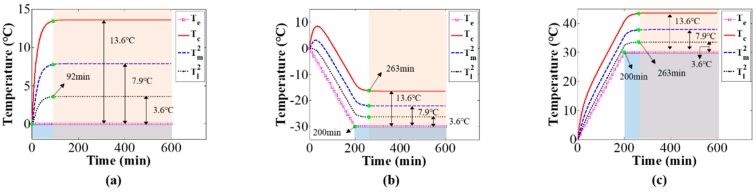
Effect of environmental temperature on the thermal equilibrium time and thermal equilibrium temperature: (**a**) $T_{e}=0$, (**b**) $T_{e}=\left\{ \begin{array}{l} -30t/200,\;0\leq t\leq 200\\ -30,\;200<t<600 \end{array}\right.$, and (**c**) $T_{e}=\left\{ \begin{array}{l} 30t/200,\;0\leq t\leq 200\\ 30,\;200<t<600 \end{array}\right.$.

Next, the influence of environmental temperature on the camera component temperature variations and distributions was investigated via simulations and experiments. The values of the thermal parameters are as given in Table 1, Table 2 and Table 3. Figure 11 shows the temperature variation of camera components with increasing environmental temperature variation rate. The simulation results indicate that when the environmental temperature changes slowly, the temperature variation values of camera component are almost the same as the environmental temperature variation values after a period of camera self-heating, and the variation of camera component temperatures can be represented by the environmental temperature variation; as the variation rate of the environmental temperature increases, the variation in camera component temperatures and the environmental temperature variation is no longer equivalent. This can explain the influence of environmental temperature variation rate on thermal-induced errors of mechanics measurement [33]; the environmental temperature variation rate affects the camera component temperature variations and distributions, and in turn affects the thermal-induced errors. Therefore, when the variation rate of environmental temperature is small, the temperature variation of the camera system can be represented by measuring the variation of environmental temperature, and then combined with the temperature compensation method [23], the thermal-induced measurement error in photomechanics can be compensated. While in the case of a large variation rate of environmental temperature, because the temperature variation of environment is not equal to that of the camera, it is necessary to calculate the temperature variation of the camera system via the proposed temperature model in this paper, and then combined with the relationship between the camera’s temperature variation and imaging parameters’ variation [33], the correction of the measurement error in photomechanics can be carried out. Figure 12 shows camera component temperature variation curves from the initial state of camera self-heating to the thermal equilibrium state for different environmental temperature variations. The simulation results show that the thermal equilibrium time and thermal equilibrium temperature of the camera components are greatly influenced by the environmental temperature and are related to the time and temperature at which the environmental temperature reached stability. On the premise that the thermal parameters remain constant, the difference value between camera-components temperature and environmental temperature is the same with different environmental temperature variation.

**Figure 13:**
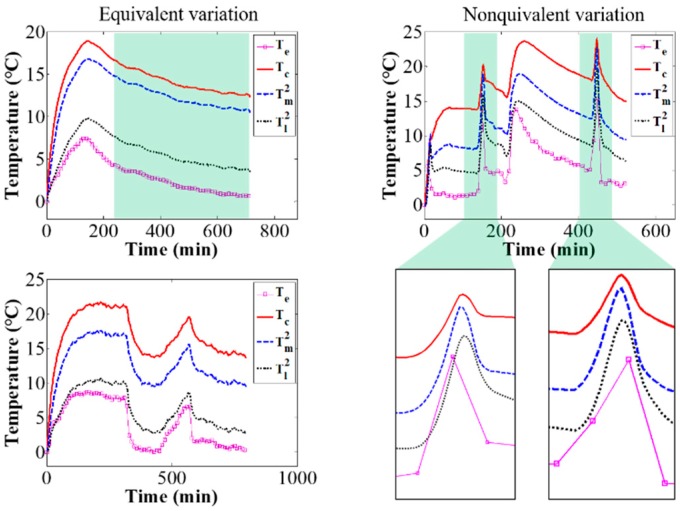
Experimental results of camera component temperatures with increasing environmental temperature variation rate.

**Figure 14:**
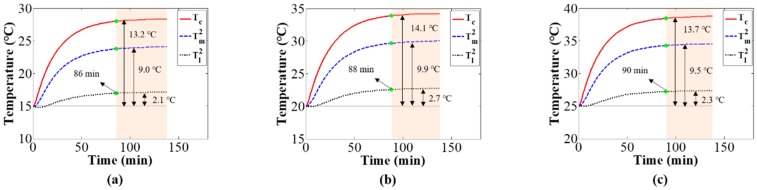
Camera component temperature variation over time under different constant environmental temperatures: (**a**) $T_{e}=15\;{}^{\circ}C$, (**b**) $T_{e}=20\;{}^{\circ}C$, and (**c**) $T_{e}=25\;{}^{\circ}C$.

Based on the experimental setup shown in Figure 4, the camera component temperatures with different environmental temperature variations were measured to verify the simulation results. Figure 13 shows the camera component temperature variations over time with increasing environmental temperature variation rate; the results are similar to those of the simulation shown in Figure 11. Figure 14 shows the working camera component temperature variations under different constant environmental temperatures (15, 20, and 25 °C). When the camera component reached thermal equilibrium, despite the difference values from camera components temperature minus environmental temperature are approximately equal for all the three experiments with different environmental temperature, there is a slight distinction for those difference values (i.e., case: 13.2, 14.1, 13.7 °C; mount: 9.0, 9.9, 9.5 °C; lens: 2.1, 2.7, 2.3 °C). The slight differences from the simulation results shown in Figure 12 can be explained as the temperature model’s thermal parameters are related to environmental temperature.

## 4. Conclusions

In this study, the heat transfer and temperature characteristics of a working digital camera were systematically investigated under the coaction of camera self-heating and environmental temperature. From the investigation results, we conclude the following: (a) Considering the time-varying environmental temperature, the temperature characteristics of a working digital camera show the heterogeneity of temperature distribution and the complexity of temperature variation, all of which are caused by heat conduction between camera’s mechanical components and heat convection between these components and the environment. (b) As the heat transfer investigations of working digital camera consider the elements of the heterogeneity of the components temperature and the time-varying environmental temperature, the temperature model proposed in this paper can describe the temperature characteristics of a working digital camera under the condition of complex environmental temperature and further obtain the camera’s thermal equilibrium time and thermal equilibrium temperature, which can be used to determine the time period of performing the mechanics measurement without thermal-induced error. (c) The thermal equilibrium time of working digital camera decreases with the increase of the heat conduction parameters and convection parameters, and increases with the increase of the specific heat parameters; the thermal equilibrium temperature of the camera is related to the heat conduction parameters and convection parameters, and has nothing to do with the specific heat parameters. So, under the premise of ensuring the optical quality, the thermal design of digital camera can be carried out by selecting camera components material with reasonable thermal characteristics (e.g., material’s thermal conductivity, surface heat transfer coefficient, and specific heat capacity) and/or changing the structural dimension of camera components (e.g., the superficial area). (d) The variation rate of environmental temperature influences the temperature characteristics of the camera. When the variation rate of environmental temperature is lower than the rate of heat transfer, the variation value of digital camera’s temperature is nearly equal to that of the environmental temperature; with the increase of the variation rate of environmental temperature, this equivalence is gradually no longer available and the temperature characteristics show that the temperature variation of digital camera lags that of environment. When it comes to thermal effect, all these conclusions can be used to guide digital camera-based mechanics measurements.

Note that considering that the investigated camera structure with internal heating source and mechanical components (i.e., camera case, mount, and lens) is universal in image-based mechanics measurement, it is reasonable to believe that the proposed temperature model and conclusions are applied to the investigation of temperature characteristics for other cameras with such structure. In addition, considering the fact that the thermal parameters of materials are related to environmental temperature, it is necessary to accurately calibrate those thermal parameters under different environmental temperature when using the proposed temperature model to investigate the camera temperature characteristics in the case of large-scale environmental temperature changes.

The results of this study provide a deeper understanding of the thermal-induced errors in image-based mechanics measurement, and are helpful to realize the unification of thermal-induced errors for indoor mechanics measurement (wherein the environmental temperature is constant) and outdoor mechanics measurement (wherein the environmental temperature changes over time). Moreover, the investigations of heat transfer processes and temperature characteristics contribute to implementing thermal design and fabrication of temperature-insensitive digital cameras.

## Figures and Tables

**Table 1:** Calibration results of Equation (3).

Parameter	*r*_1_	*r*_2_	*r*_3_	*r*_4_	*r*_5_	*r*_6_	*k*_1_	*k*_2_	*k*_3_	*k*_4_
value	0.21	0.03	0.09	0.06	0.03	0.21	0.76	0.17	0.97	0.87

**Table 2:** Calibration results of Equation (5).

Parameter	*k_m_*	*m_m_*
value	143.80	0.13

**Table 3:** Calibration results of Equation (6).

Parameter	*k_l_*	*m_l_*
value	116.41	0.55

**Table 4:** Values of heat conduction parameters.

Parameter	*r*_1_	*r*_3_	*r*_5_	*k_m_*	*k_l_*
I	0.17	0.07	0.02	115.04	93.13
II	0.21	0.09	0.03	143.80	116.41
III	0.25	0.11	0.04	172.56	139.69

**Table 5:** Values of heat convection parameters.

Parameter	*r*_2_	*r*_4_	*r*_6_	*m_m_*	*m_l_*
I	0.02	0.05	0.17	0.10	0.44
II	0.03	0.06	0.21	0.13	0.55
III	0.04	0.07	0.25	0.16	0.66

**Table 6:** Values of specific heat parameters.

Parameter	*k*_1_	*k*_2_	*k*_3_	*k*_4_	*n_m_*	*n_l_*
I	0.61	0.14	0.78	0.70	0.80	0.80
II	0.76	0.17	0.97	0.87	1.00	1.00
III	0.91	0.20	1.16	1.04	1.20	1.20
